# Bile composition in Alagille Syndrome and PFIC patients having Partial External Biliary Diversion

**DOI:** 10.1186/1471-230X-8-47

**Published:** 2008-10-20

**Authors:** Karan M Emerick, Marc S Elias, Hector Melin-Aldana, Sandra Strautnieks, Richard J Thompson, Laura N Bull, AS Knisely, Peter F Whitington, Richard M Green

**Affiliations:** 1 Children's Memorial Hospital, Chicago, IL, USA; 2 Northwestern University Feinberg School of Medicine, Chicago, IL, USA; 3 Department of Liver Studies and Transplantation, King's College London School of Medicine, London, UK; 4 Department of Medicine, San Francisco General Hospital, San Francisco, CA, USA; 5 Institute of Liver Studies, King's College Hospital, London, UK

## Abstract

**Background:**

Partial External Biliary Diversion (PEBD) is a surgical intervention to treat children with Progressive Familial Intrahepatic Cholestasis (PFIC) and Alagille syndrome (AGS). PEBD can reduce disease progression, and examining the alterations in biliary lipid composition may be a prognostic factor for outcome.

**Methods:**

Biliary lipid composition and the clinical course of AGS and PFIC patients were examined before and after PEBD.

**Results:**

Pre-PEBD bile from AGS patients had greater chenodeoxycholic/cholic acid (CDCA/CA), bile salt, cholesterol and phospholipid concentrations than PFIC patients. AGS patients, and PFIC patients with familial intrahepatic cholestasis 1 (FIC1) genotype, responded better to PEBD than PFIC patients with bile salt export protein (BSEP) genotype. After successful PEBD, AGS patients have higher biliary lipid concentrations than PFIC patients and PEBD also increases biliary phospholipid concentrations in FIC1 patients.

**Conclusion:**

Both AGS and FIC1 patients can benefit from PEBD, and preserved biliary phospholipid concentrations may be associated with better outcomes post-PEBD.

## Background

Genetic liver diseases are common causes of severe cholestasis and progressive liver disease in children. Alagille Syndrome (AGS) is a systemic disease caused by heterozygous mutations in the Jagged 1 gene (*JAG1*) or Notch2 gene (*NOTCH2*) [1,2]. Progressive familial intrahepatic cholestasis (PFIC) comprises a group of autosomal recessive diseases [3]. Two commonly recognized forms of PFIC are associated with relatively low serum GGT [4]: 1) familial intrahepatic cholestasis 1 (FIC1) disease caused by mutations in *ATP8B1 *[5] and; 2) bile salt export protein (BSEP) disease caused by mutations in *ABCB11 *[6]. The pathophysiology of cholestasis differs among these disorders. AGS is associated with a paucity of interlobular bile ducts while PFIC is associated with defects in bile transporters.

Partial external biliary diversion (PEBD) has been used to treat PFIC and AGS [7-9]. PEBD involves the surgical placement of an enteric conduit between the gallbladder and the skin through which bile flow is partially diverted away from the enterohepatic circulation, resulting in an approximately fifty percent diversion of bile flow [10]. PEBD has been effective in improving chronic cholestasis and its associated complications. PEBD is not definitively known to alter the progression of liver disease in AGS, which progresses to cirrhosis in 20–40% of patients. In contrast, PEBD has become a standard intervention for PFIC, which otherwise progresses to cirrhosis and end-stage liver disease [4,7,10-13]. PEBD may not always affect the natural progression of either AGS or PFIC, and no clinical parameters have been defined that predict patients likely to respond to PEBD.

Biliary lipids include bile salts, cholesterol and phospholipid. The relative concentrations of hydrophobic to hydrophilic bile salts is also a major determinant of the ability to form mixed micelles to maintain the stability of lipids in suspension. Little is known regarding biliary lipid composition in genetic cholestasis or the effects of PEBD, and mouse models of PFIC and AGS unfortunately differ from the human diseases, likely because the murine bile salt pool is highly hydrophilic [14,15]. Careful analysis of biliary lipid composition in patients with AGS and PFIC may improve our understanding of the pathophysiology and treatment of these conditions. We analyzed the biliary lipid composition in patients with AGS, FIC1 disease and BSEP disease before and after PEBD to determine baseline levels, and to determine if composition is predictive of clinical response to PEBD.

## Methods

### Human Subjects

Patients with PFIC or AGS who underwent PEBD at Children's Memorial Hospital, Chicago, IL and the Children's Hospital of Philadelphia, Philadelphia, PA between 2000 and 2004 constituted the study population. The diagnosis of AGS was made by a liver biopsy documenting paucity of interlobular bile ducts in association with at least three other major features of AGS [16]. The diagnosis of PFIC was made by the presence of chronic progressive cholestasis in association with low serum GGT [4]. FIC1 and BSEP disease in the subjects with PFIC were determined by genetic analysis. Indications for PEBD in AGS patients included incapacitating pruritus and/or xanthomatosis refractory to medical therapy, whereas in PFIC it was performed as primary treatment. All patients had been administered medical therapy consisting of ursodeoxycholic acid in doses of 20 mg/kg without success, as defined by persistent debilitating pruritus. Clinical data and bile were collected prospectively and the degree of pruritus was assessed on a four-point scale [8]. The pruritus score is derived from a combination of patient (when capable) and parent reports of scratching, the observed behavior of the child and the child's physical findings. So that 0 = no scratching, 1 = mild scratching when undistracted without abrasions, 2 = active scratching without abrasions, 3 = active scratching and abrasions, 4 = cutaneous mutilation with bleeding and scarring from scratching.

Bile samples were obtained intra-operatively from gallbladder bile during PEBD and post-operatively from output into ostomy bags. The post-operative collections were collected first thing in the morning, before a meal, from the bile that accumulated overnight. Informed consent in writing was obtained by the parents or legal guardian of the patients. The study protocol conformed to the ethical guidelines of the 1975 Declaration of Helsinki and had approval of the Institutional Review Board.

Before data analysis, patients were divided into the categories of clinical "responder" versus "nonresponder" by these criteria: 1) In "responders", serum bile salt concentrations fell below 40 μM/L and/or pruritus score fell ≥2 points; 2) "nonresponders" maintained a serum bile salt concentrations ≥40 μM/L and/or had ≤ 1 point fall in pruritus, or progressed to end-stage liver disease or received liver transplantation.

### Histological Assessment of Liver Pathology

Microscopy of liver biopsy samples obtained before and/or at the time of PEBD were analyzed by a blinded liver pathologist. Sections were stained with hematoxylin/eosin and Masson's trichrome. Portal-tract fibrosis, pericellular fibrosis, parenchymal changes (giant-cell transformation, rosetting and pseudoacinus formation, ballooning of hepatocytes) and degree of cholestasis were assessed as previously described [17].

### Mutational analysis, patients with PFIC

Peripheral blood leukocytes from patients diagnosed with PFIC and from their parents were collected, with DNA extracted and evaluated as described [5,18,19]. Children of Amish origin with extended-family members known to carry the 923G>T (G308V) "Byler mutation" in *ATP8B1 *[18] were assessed for homozygosity for this nucleotide substitution. *ABCB11 *was sequenced in patients without an *ATP8B1 *mutation who lacked BSEP expression on immunohistochemical study of livers [19], with confirmation of patient mutations by analysis of parental DNA.

### Analysis of biliary lipid composition

The concentrations of bile salts, cholesterol and phospholipids were measured in bile that was obtained from patients and stored at -80°C. Stoma bag contents reflected 1–4 hours worth of bile excretion. There was no evidence of deconjugation of bile salts in the analyzed samples, indicating that significant metabolism of bile by bacteria was unlikely. Measurements were performed in triplicate. Bile salt species were determined by High Performance Liquid Chromatography according to the method of Carey and Armstrong [20]. Bile salts were expressed as ratios of taurine to glycine bile salt conjugates (T/G) and chenodeoxycholic acid (CDCA) to cholic acid (CA) species (CDCA/CA). Bile salt hydrophobicity index (HI) was calculated according to the method of Heuman [21]. Following a Folch extraction, bile cholesterol content was performed using a spectrophotometric assay (Sigma, St. Louis, MO) and bile phospholipid content was assessed using the method of Bartlett [22]. The percent change in serum chemistry value per patient post-PEBD is calculated as (post-PEBD – pre-PEBD/pre-PEBD) × 100).

### Statistics

Statistical analysis was performed using SPSS for Windows (SPSS, Chicago, IL). Data are presented as median and ranges. Comparisons of baseline characteristics among groups were performed using the Kruskal-Wallis test and comparisons within groups before and after PEBD were performed using the Mann-Whitney U test.

## Results

Three unrelated AGS patients and seven children with PFIC were identified and consented for the study. The PFIC patients included four Amish children from the same family with FIC1 disease homozygous for the 923G>T (G308V) mutation in *ATP8B1*; and three unrelated children with BSEP disease that carried two *ABCB11 *mutations that would disrupt BSEP synthesis or function. The sequenced mutations included: patient 8 (611+1 G>A/890 A>G (heterozygote), E297G); patient 9 (IVS13del-13^-8/890 A>G (heterozygote), E297G) and patient 10 (1460 G>C (homozygote), R487P). The patients were 7.5 ± 6.4 years old (range 0.4 – 18) at the time of PEBD. The 3 AGS patients were older than the PFIC patients (15.2 ± 3.27 vs. 3.5 ± 2.49 years, p < 0.001), whereas FIC1 disease and BSEP disease patients received PEBD at similar ages (4.25 ± 2.8 vs. 2.53 ± 2.0 years). The follow-up bile samples were collected at 1.35 ± 0.75 years after PEBD (range 0.3–2.5 years).

### Clinical criteria and response to PEBD

Patients had elevated serum ALT (97, 40 – 265 IU/L) and bilirubin (4.4, 1.2 – 10.8 mg/dL) concentrations, and marked pruritus before PEBD (Table 1). All patients had elevated serum bile acid levels before PEBD (183, 58 – 485 μM/L) with PFIC patients having significantly higher levels (254, 97 – 485 μM/L) than AGS (81, 58 – 97 μM/L) (p = 0.01). Pruritus was slightly greater in AGS than PFIC patients. FIC1 and BSEP disease patients did not differ in regard to serum bilirubin, ALT, bile salts or level of pruritus. There was no difference in the pre-PEBD serum bilirubin level, ALT level, bile salt levels or degree of pruritus between patients who responded and those that did not respond.

**Table 1:** Clinical Data OF Patients with AGS, FIC1 and BSEP Disease

			**Pre-PEBD Baseline**					**Post-PEBD**				
**Pt**	**Disease**	**Age at PEBD** **(yrs)**	**Itch**	**T Bili** **(mg/dL)**	**GGT** **(IU/L)**	**ALT** **(IU/L)**	**Bile salts** **(μM/L)**	**Age at F/U** **(yrs)**	**Itch**	**T Bili** **(mg/dL)**	**ALT** **(IU)**	**Bile salts** **(μM/L)**

1	AGS	11.6	4	1.2	170	82	81	14	1	2.1	198	24
2	AGS	18	4	4.4	370	265	58	18.3	1	2.8	155	24
3	AGS	16	4	2.0	329	121	97	16.8	2	2.9	214	21
4	FIC1	0.5	3	8.9	32	97	97	1.5	1	0.6	270	6
5	FIC1	4.4	4	5.4	33	53	255	6.1	1	0.3	74	6
6	FIC1	4.8	4	10.8	23	40	340	5.8	1	6.9	68	120
***7***	FIC1 *	***7.3***	***4***	***6.7***	***28***	***52***	***485***	***9***	***4***	***4.5***	***28***	***63***
***8***	BSEP*	***0.4***	***3***	***3.1***	***45***	***112***		***2.9***	***2***	***2***	***83***	***488***
***9***	BSEP*	***2.7***	***3***	***4.7***	***29***	***190***	***184***	***4.3***	***4***	***4.5***	***171***	***257***
10	BSEP	4.5	2	4.1	79	173	225	5	1	0.8	85	32

T Bili = Total Bilirubin. Non-responders*.

Patients had a significant decrease in serum bilirubin (2.8, 0.3 – 6.9 mg/dL) and pruritus score (2, 1 – 4)) after PEBD. The serum bile acids post-PEBD were significantly lower with median 24 (21 – 488) μM/L, with the AGS group being median 24 (21 – 24) μM/L and PFIC group being median 62 (6 – 488) μM/L. In PFIC responders, the bile acid level was significantly lower (6.0, 6.0 – 32 μM/L) than both pre-PEBD levels and the post-PEBD levels of non-responders (119, 62 – 488 μM/L). AGS patients had a significant decrease in pruritus. The serum CDCA/CA ratio post-PEBD was not statistically different among diseases, or among responders versus non-responders.

Overall, three of the 4 FIC1 disease patients had successful clinical responses to PEBD. The oldest at PEBD (patient 7) did not respond; 1.7 years after PEBD he had no improvement in pruritus and had evidence of advancing liver disease with portal hypertension. One of the 3 BSEP disease patients responded to PEBD while the other two (patients 8 and 9) did not respond.

### Histopathologic findings

BSEP disease patients overall had the most aggressive histological changes with prominent pseudoacinar changes and portal fibrosis (Table 2). Of the PFIC patients with portal fibrosis grade ≥ 3, one of three were responders, while of those with fibrosis grade <3, three of four were responders. Of the 3 BSEP disease patients, the responder had grade 2 portal fibrosis, whereas the two non-responders had grade 2 and 4 fibrosis. Only one AGS patient had undergone biopsy at the time of PEBD (patient 1).

**Table 2:** Liver Histology prior to Partial External Biliary Diversion

		Fibrosis		Parenchyma		
Pt	Disease	Portal	Pericellular	Cholestasis	Pseudoacini	Ballooning

1	AGS	2	1	2	0	0
2	AGS	NA	NA	NA	NA	NA
3	AGS	NA	NA	NA	NA	NA
4	FIC1 disease	2	1	0	0	0
5	FIC1 disease	3	3	1	0	0
6	FIC1 disease	2	1	3	0	2
***7***	***FIC1 disease***	***3***	***1***	***2***	***1***	***0***
***8***	***BSEP disease***	***4***	***1***	***2***	***2***	***0***
***9***	***BSEP disease***	***2***	***1***	***3***	***1***	***1***
10	BSEP disease	2	2	3	0	0

Non-responders are in **bold **and *italics*

NA = not available because liver biopsy not performed at time of PEBD.

### Lipid composition of bile

**Figure 1:**
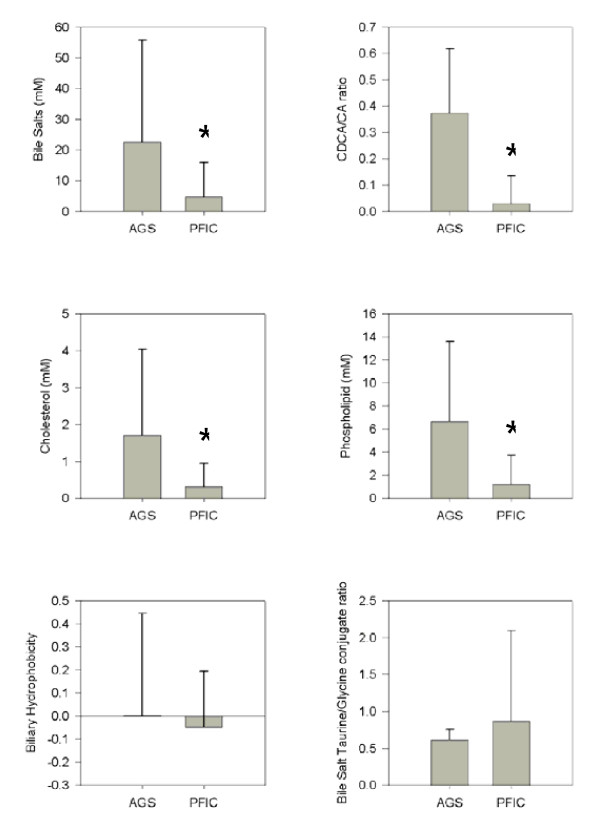
**Biliary lipid content in AGS and PFIC patients prior to Partial External Biliary Diversion (PEBD)**. Gallbladder bile was obtained from patients prior to PEBD and analyzed for bile salt, cholesterol and phospholipid content. AGS (n = 3), PFIC (n = 7). Mean ± SD. * p < 0.05 vs AGS.

The bile from PFIC patients had significantly lower concentrations of all biliary lipids (bile salts, cholesterol and phospholipids) and a lower ratio of chenodeoxycholic acid (CDCA) to cholic acid (CA) than AGS patients (Figure 1), (p < 0.05). The ratio of taurine to glycine bile salt conjugates in bile and the hydrophobicity index was not different between PFIC and AGS patients.

Bile from FIC1 disease and BSEP disease patients were not different in regard to total bile salt, cholesterol and phospholipid content. They differed significantly, however, in the percent ursodeoxycholic acid enrichment and hydrophobicity. BSEP disease patients had median 37, range 29 to 60% ursodeoxycholic acid enrichment compared to median 2.29, range 0.9 to 4.2 in FIC1 disease patients (p = 0.05). As a result, the hydrophobicity index of bile was median -0.15, range -0.05 to -0.26 in BSEP disease patients vs. median 0.035, range 0.001 to 0.07 in FIC1 patients (p= 0.02). The pre-PEBD biliary lipid composition of PFIC patients who responded to PEBD was not significantly different from non-responders.

**Figure 2:**
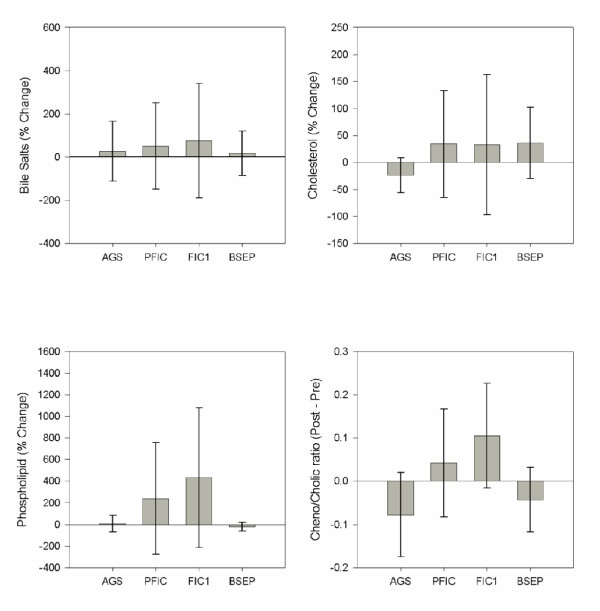
**Change in lipid content of bile resulting from Partial External Biliary Diversion (PEBD)**. Bile was obtained from patients before and after PEBD and analyzed for bile salt, cholesterol and phospholipid content. The data is presented as the percent change in biliary lipid content. AGS (n = 3), PFIC (n = 7; FIC1: n = 4 and BSEP n = 3) * p < 0.05 vs pre-PEBD levels.

**Figure 3:**
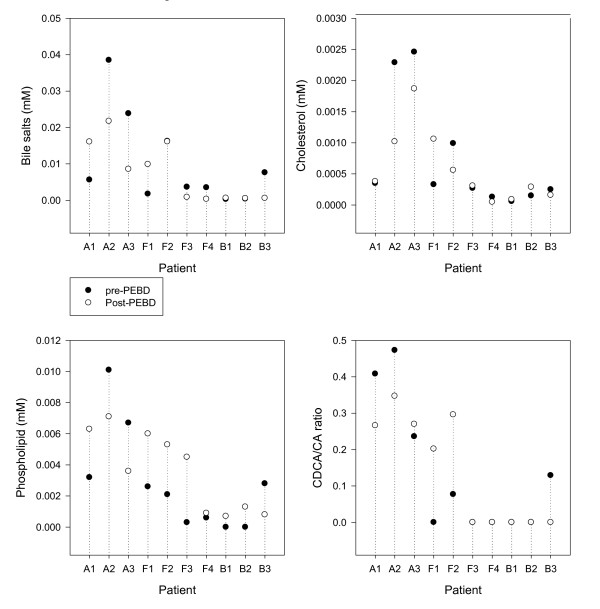
**Change in lipid content of bile after Partial External Biliary Diversion (PEBD) in Individual Patients**. A = AGS, F = FIC1, B = BSEP. Bile was obtained from PFIC (n = 7) patients before and after PEBD who had been independently classified as Responders (n = 4) or Non-Responders (n = 3) based on clinical criteria. Patients identified as non-responders were F3, B1 and B3.

The data regarding the percent change in biliary lipid composition after PEBD are presented in Figure 2 and the individual data in Figure 3. AGS patients showed no significant change in biliary lipid composition after PEBD. Patients with FIC1 disease showed a 4-fold increase in biliary phospholipid content after PEBD (median 1.3, range 0.3 to 2.6 mM, to median 4.9, range 0.9 to 6.0 mM, p = 0.05), and a trend towards an increased CDCA/CA ratio (median 0.00, range 0.00 to 0.07 to median 0.10, range 0.00 to 0.29, p = 0.10). BSEP patients had no significant increase in biliary lipids and had no detectable biliary CDCA post-PEBD, resulting in a decreased CDCA/CA ratio (median 0.00, 0.00 to 0.13 to median 0.0, range 0.0 to 0.0).

PFIC responders showed a trend toward increased bile salt concentration and CDCA/CA ratio in bile after PEBD, although these did not reach statistical significance (Figure 3), but no increase in cholesterol and phospholipid content. In 2 of 3 PFIC non-responders there was a complete absence of CDCA in bile after 1 year of diversion. AGS patients continued to have higher biliary lipid concentrations after PEBD than PFIC patients, both responders and non-responders.

### Long-term outcomes after successful PEBD

Patients 4 and 5 had the best outcomes, with relief of cholestasis and marked reduction in serum bilirubin levels. Patient 6 developed severe pancreatitis associated with multi-organ system failure and death 2 years after PEBD. Patient 7 died shortly after liver transplantation. Patient 8 underwent successful liver transplantation 2.5 years after PEBD for massive ostomy output, and patient 9 developed hepatocellular carcinoma 1.6 years post-PEBD and died (previously reported in reference 19). Thus, only two of this group of PFIC patients survived with PEBD to demonstrate long-term benefit. All three AGS patients have had good long-term outcome.

## Discussion

Biliary lipid compositions of children with PFIC and AGS remain poorly understood, and these data demonstrate that they differ according to disease. The biliary lipid composition of AGS patients was essentially normal, whereas bile from PFIC patients contained minimal biliary lipids. This likely reflects the differing pathophysiology of the diseases. The bile from PFIC patients with FIC1 genotype and BSEP genotype both had very low lipid concentrations but were not significantly different from each other. This may reflect similar mechanisms of cholestasis in both types of low-GGT PFIC, with a primary impairment of transporter expression and function. It should be noted that this study examined lipid concentrations in bile, rather than secretion rates. In addition, pre-PEBD bile was obtained from the gallbladder, while post-PEBD bile was obtained from the stoma.

After clinically successful PEBD, the bile composition of FIC1 patients had a four-fold increase in phospholipid content without changes in bile salts or cholesterol. The bile remained relatively normal in AGS patients and no significant changes were noted in BSEP patients. Lipid content of all PFIC patients who responded to PEBD demonstrated a trend to increased bile salt, cholesterol and CDCA/CA content after PEBD, although these did not reach statistical significance. There were a limited number of patients with these rare diseases and significant variability between the human subjects, so one cannot exclude the possibility that increases in the concentrations of these lipids could not be detected in this study. Also, given that there was significant homogeneity in the FIC1 patients who shared the same mutation compared to the BSEP patients who were unrelated with distinct mutations, it is possible that the specific mutation in the FIC1 patients may intrinsically be associated with a milder disease and more favorable outcome in this study. We were unable to identify any other parameters such as age at diversion, histological stage or liver function levels before diversion that served as prognostic indicators for success in PFIC patients undergoing PEBD.

Baseline bile lipid content at PEBD differed in composition between AGS patients and PFIC patients; differences likely reflecting differences in the etiology of cholestasis [23]. In AGS, the paucity of interlobular bile ducts (fewer than 0.4 bile ducts per portal area) [24] is believed to underlie cholestasis by causing obstruction of biliary drainage. Sixty to eighty percent of hepatic lobules lack biliary drainage while remaining capable of producing bile, more in the periphery than the core [25]. AGS patients have serum values characteristic of biliary obstruction with markedly elevated serum cholesterol, bile salts, and lipoprotein-X values, presumably due to reflux of bile constituents from canaliculi and ductules with inadequate bile drainage [26-28]. Our findings suggest that patients with AGS make normal bile similar to that reported for normal children [29]. Thus, it appears that cholestasis in AGS is due to excreting too little "normal bile" or retaining too much. The ability of AGS patients' hepatocytes to make and secrete bile may protect them from cellular cholestasis and cholate injury, and provides a putative explanation why progressive liver disease is relatively uncommon [30].

Compared to AGS bile, PFIC bile contains very low concentrations of lipid. This finding reflects the functions of the PFIC gene products, FIC1 and BSEP, which are pivotal in canalicular bile formation. The potentially resultant effect on PFIC serum levels is a relatively lower cholesterol concentration and absence of lipoprotein-X [31,32]. The low molar ratio of CDCA/CA in PFIC bile likely reflects the relatively poor excretion of hydrophobic bile salts [7,33], which is evidenced by the preponderance of CDCA in the serum of these patients [4,34,35], although a causative relationship remains speculative.

The bile composition of AGS patients does not change after successful PEBD. Dramatically lower serum cholesterol values in AGS patients after successful PEBD [9] probably reflect shunting of cholesterol to bile acid synthesis, similar to the effect of oral bile acid binding resins on serum cholesterol in hypercholesterolemia [36]. In AGS, PEBD may simply shunt bile away from the enterohepatic circulation, without fundamentally changing primary hepatobiliary function.

## Conclusion

In this study, we relate the outcome of PEBD and the results of biliary lipid analysis to the specific subsets of genetically defined PFIC. In FIC1 patients, there was an increase in phospholipid content and trend toward an increase in CDCA/CA, although the latter affect did not reach statistical significance. A positive clinical response to naso-biliary drainage has been observed in patients with benign recurrent intrahepatic cholestasis (BRIC), also due to a defect in FIC1 [37].

In conclusion, our study confirms that the biliary lipid concentration in patients with AGS is higher than in patients with PFIC. In addition, PEBD can relieve cholestasis in AGS without changing bile composition; and improves phospholipid content in the bile of FIC1 patients. Larger scale comparisons of these populations with consideration for the effects of specific gene defects on outcome of PEBD will be necessary before definitive conclusions of prognosis can be derived.

## Competing interests

The authors declare that they have no competing interests.

## Authors' contributions

KE designed the study, recruited the patients, collected and analyzed the samples, performed the statistical analysis and drafted the manuscript. ME carried out the sample analysis and participated in the interpretation and analysis of the data. HMA reviewed the liver biopsies and developed the grading scale used in quantifying histological changes for the analysis and helped to revise the manuscript. SS, RT and LB carried out the molecular genetic studies as well as participated in the drafting and revising of the manuscript for important intellectual content. AK made substantial contributions to the analysis and interpretation of the data. PFW designed the study with KE and was the leader in the conception of the project, its design and ultimately the interpretation of the results. RG supervised the study, provided his laboratory equipment, supplies and technical help to perform the bile analysis and made substantial contributions to the acquisition, analysis and interpretation of the data as well as in drafting the manuscript. All authors read and approved the final manuscript.

## Pre-publication history

The pre-publication history for this paper can be accessed here:


